# Evaluation of the demographic characteristics and general health status of earthquake survivors affected by the 2023 Kahramanmaraş earthquake; a section from Gaziantep Nurdağı district

**DOI:** 10.1186/s12889-024-18444-7

**Published:** 2024-04-01

**Authors:** Leman Tomak, Tolga Demirel, Ibrahim Demir

**Affiliations:** 1 School of Medicine, Department of Biostatistics and Medical Informatics, Ondokuz Mayis University, 55200 Samsun, Turkey; 2 Turkish Statistical Institute, Gaziantep Recional Office, Degirmicem Mah. Sehit Murat Yasilak Sok. No:13/A, Gaziantep, Turkey; 3 Turkish Statistical Institute, Devlet Mah. Necatibey Cad. No:114 Cankaya, Ankara, Turkey

**Keywords:** Natural disaster, Earthquake, Earthquake victims, Individual behaviour, Trauma, Injury, Fractures, Mental health, General Health Questionnaire (GHQ-12)

## Abstract

**Background:**

An earthquake with a magnitude of 7.7 occurred in Pazarcık District of Turkey at 04.17 on February 6, 2023 and another earthquake of 7.6 occurred at 13.24 on the same day. This is the second largest earthquake to have occurred in Turkey. The aim of this study is to investigate the earthquake-related level of knowledge, attitudes and behaviours, general health and psychological status of survivors who were affected by the 2023 Kahramanmaraş Earthquake and who were living in Nurdağı District of Gaziantep after the earthquake.

**Methods:**

Data of 2317 individuals older than 18 years of age who were living in earthquake neighbourhoods, tents and containers in Nurdağı District of Gaziantep were examined. Variables were evaluated to find out the demographic characteristics and general health status of earthquake victims. General Health Questionnaire (GHQ-12) was used to find out psychological states of earthquake victims.

**Results:**

The rate of injuries was 14.2% and leg and foot injuries were the most common with 44.2%. The relationship between injury status; and age, marital status, and being trapped under debris was revealed (*p* < 0.05). Mean GHQ-12 score of the survivors was 3.81 ± 2.81 and 51.9% experienced psychological distress. In the evaluation with logistic regression, it was found that female gender, being injured in the earthquake, loss of first degree and second degree relatives (with a higher rate in loss of first degree relative), having a severely damaged -to be demolished house and having a completely destroyed house were correlated with higher level of psychological distress (*p* < 0.05).

**Conclusion:**

General characteristics, injury prevalence and affecting factors of earthquake survivors were evaluated in the present study. Psychological distress was found in victims. For this reason, providing protective and assistive services to fight the destructive effects of earthquake is vital. Accordingly, increasing the awareness of people residing in earthquake zones regarding earthquakes is exceptionally important.

## Background

Earthquakes are sudden, large-scale natural disasters that lead to destruction in wide ranges [1]. They impair public health both physically and mentally to an important extent partly because of their unpredictability and destructiveness [2]. The earthquakes occurred in the past century took away millions of lives and injured thousands of individuals. The top priority of the medical sector after an earthquake is to reduce mortality and battle psychological morbidity at the earthquake site. For this reason, the analytical examination of the physical and psychological health problems afflicting earthquake victims carries significant weight in the evaluation of earthquake models [3].

Injuries are seen as the most prominent reason for morbidity and primarily include soft-tissue or orthopedic injuries. These injuries are more often present in the earthquake victims’ extremities, especially in the lower extremities, and tend to be infected. Crush injuries can potentially escalate into fatal crush syndrome [4, 5]. Among other forms of injury are ischemic cardiovascular diseases, hypertension, and respiratory diseases that are associated with the inhalation of particulate matter [1, 3]. At the same time, acute kidney injury, hemorrhages, and outbreaks of infectious diseases are other important health problems that demand attention [1, 4].

Previous studies have generally shown that earthquake survivors have increased psychological symptoms after the earthquake and are more susceptible to psychological illnesses, particularly post-traumatic stress disorder (PTSD), thereafter. Several studies investigating the correlation between psychological morbidity and earthquake occurrence have been reported following earthquakes around the world [6]. Approximately 70% of all earthquake survivors are confronted with traumatic incidences and post-traumatic stress symptoms in their lifetime [7]. General Health Questionnaire (GHQ) is extensively used to identify the psychological condition of earthquake survivors as well as the associated factors in order to evaluate the earthquake survivors’ state of health. This survey which signals the poorness of the respondent’s health through high scores received on the scale is a short, simple, and easily applied tool [6]. At the same time, after experiencing earthquake trauma, the risk of mental disorders being aggravated increases, stress sensitivity increases, and cognitive decline may occur; in fact, brain functions and structure may deform [8].

Understanding how earthquake victims behave in the event of an earthquake is crucial in earthquake risk management. Meaningful earthquake risk knowledge helps judge the earthquake risk scenarios so that correct decisions can be made about the measures and adjustments to diminish the impact of the earthquake, at a faster pace [9].

In Turkey, an earthquake of 7.7 magnitude occurred in Pazarcık District of Kahramanmaraş Province on 6 February 2023 at 04.17 a.m; on the same day, another earthquake of 7.7 magnitude occurred in Elbistan District of Kahramanmaraş Province at 1.24 p.m. These earthquakes are the biggest earthquakes to take place in Turkey after the 1999 İzmit Earthquake. This earthquake was felt intensely in the surrounding provinces such as Gaziantep, Hatay, Osmaniye, Adıyaman, Şanlıurfa, Diyarbakır, Malatya, Adana, and several others. In Gaziantep Province, in Araban, Islahiye, Karkamış, Nizip, Nurdağı, Yavuzeli, and central districts, 3913 people lost their lives whilst 37,238 houses were damaged [10]. In particular, doublet earthquakes that occur on the same day are significantly more destructive, and their impact on survivors is overwhelming [5].

The sufficiency and analysis of the data accumulated to put forth the injury and health conditions encountered in big earthquakes are of great importance. They allow models pertaining to the earthquake to be established, earthquake preparation kits to be developed, and standards to be defined [3]. The evaluation of the knowledge, attitude, and behavior of earthquake survivors; the impact of these measures on the status of injury as well as the status of mental health; and the evaluation of the relationship between these measures and the demographic features of survivors are important topics.

In this study, our aim was to present the knowledge, attitude and behavior of the earthquake victims and earthquake survivors in Gaziantep Nurdağı and to evaluate their general health and psychological conditions, as well as the factors affecting these conditions in the 2023 Kahramanmaraş Earthquake.

## Methods

### Study design and participants

In this study, data concerning the demographic features, knowledge, attitude, behavior, general health, and psychological conditions of citizens living in the Nurdağı District of Gaziantep Province were evaluated after the earthquakes that occurred on 6 February 2023 in the Pazarcık and Elbistan Districts as well as the ongoing aftershock earthquakes in Gaziantep Province. Ethical approval for this study was obtained from Medical Research Ethical Committee of a university (decision number 2023/134).

The fieldwork for the data received from Turkish Statistical Institute Gaziantep Regional Office took into account the citizens living in tents and containers built in the Nurdağı District, as well as the citizens residing in rural neighborhoods like Gedikli, Gökçedere, İkizkuyu, Kırışkal, Naimler, Sakçagözü, Şatırhüyük, and Terken; while the sample size was calculated as the inclusion of at least 2208 people in the study, with a 2% error at the 95% confidence limit, out of 27,383 earthquake victims, and the data of 2317 people over the age of 18 were included in the study.

The general and quantitative data for this study related to the earthquake survivors residing in the Nurdağı District of the Gaziantep Province were acquired from the District Governorship of Nurdağı. The population data concerning the Nurdağı District in 2022 were acquired from the Turkish Statistical Institute and the sample size was calculated with the ratio of earthquake victims to the population. It was decided to conduct a survey with 992 earthquake victims in tent and container cities, and 1,325 in neighborhoods outside the district center. For the survey, earthquake neighborhoods, tents and containers were determined by systematic sampling method by listing them according to their 2022 population size. Quotas were created for gender and age groups, taking into account the demographic characteristics of the 2022 population data of Nurdağı District. Surveyors were enabled to carry out surveys within the framework of this quota, starting from different points of the. residential area.

The criterion for being included in the study was being older than 18 years old while the criteria for being excluded from the study was being younger than 18 years old, not being in the Nurdağı District during the Kahramanmaraş Earthquake, and being bedridden. The field study was administered by 23 survevors, 1 sociologist, and 3 statisticians between the 10th and 19th of April in 2023. All earthquake victims included in the sample were interviewed and the surveys were compiled using the computer-assisted face-to-face interview method (CAPI).

A pilot study intended to prepare and test the survey questions was implemented before starting the research process. Survey questions were prepared in line with the information obtained by face-to-face interviews with earthquake victims in tent and container cities in Nurdağı district and in Sakçagözü Neighborhood, and then they were tested in these settlements.

The study data were collected from 2317 individuals above the age of 18. The participants had an average age of 45.71 ± 15.62 (18–90) years; 50.7% of the participants were men and 49.3% of the participants were women. Married participants constituted 82.1% and housewives constituted 42.1% of all participants. Other general features regarding the participants are illustrated in Table 1.

**Table 1:** General characteristics of the study group (*n* = 2317)

		n	%
**Age**			
	≤ 20	99	4.3
	21–30	370	16.0
	31–40	475	20.5
	41–50	492	2.2
	> 50	881	38.0
**Gender**			
	Male	1174	50.7
	Female	1143	49.3
**Marital status**			
	Single	228	9.8
	Married	1903	82.1
	Divorced/widowed	186	8.0
**Educational level**			
	No degree	526	22.7
	Below high school	1308	56.5
	High school	336	14.5
	Above high school	147	6.3
**Employment status**			
	Employed	871	37.6
	Housewife	975	42.1
	Student	47	2.0
	Unemployed	116	5.0
	Retired/ill/old	308	13.3
**Place of residence**			
	Neighbourhood	92	4.0
	Tent	1055	45.5
	Container	1170	50.5

### Measurements

In this study, the variables used to determine the demographic features and general health conditions of earthquake victims were evaluated. Whilst the earthquake victims’ knowledge of earthquakes before the earthquake, experiences during the earthquake, employment status, general health conditions, and living conditions after the earthquake were investigated; their age, education status, marital status, work-life before the earthquake, and housing occupancy status after the earthquake were assessed. The cases of being trapped under the debris, being injured, or having injured family members, losing a family member, and the state of general health evaluations were put forth and their impact on the model was interpreted.

Besides the scalings and codes employed by the Turkish Statistical Institute (TSI) in its own survey-based studies, the scaling was made with respect to the descriptions of the buildings in the district determined by the Disaster and Emergency Management Presidency of Turkey (AFAD) and the Nurdağı District Governorship when gathering data. The categorization and coding of the variables were based on the procedures and principles specified by the ‘’Statistical Classifications Regulations’’ published on the 28th of June 2020 by the Methodology Department of the TSI in the duplicate official newspaper numbered 31,022.

The 12-item General Health Questionnaire (GHQ-12) was used for psychological distress measurements to evaluate the mental health of earthquake victims. The questionnaire was developed by David Goldberg to define acute mental disorders that are common in the society [11, 12]. Validity and reliability study of the questionnaire was conducted by Kılıç in Turkey [13].

This screening instrument, which was used for the psychological evaluation of earthquake victims in the last several weeks, examines variables like insomnia, difficulty with everyday tasks, inattentiveness, inability, decision-making, coping skills, happiness, enjoyment, confidence, depression, and feeling of worthlessness. The options provided to respondents for these variables are as “not at all”, “as usual”, “more than usual”, and “very often” in this easily applied and practical screening instrument. In the GHQ-12 scoring, while many different methods like the original (0-0-1-1) method (GHQ), corrected scoring (0-1-1-1), and Likert (0-1-2-3) scoring are available, the original method developed by Goldberg was used in this study [14–16]. In the study, the average GHQ score was taken as the cutoff point, and earthquake survivors were separated into two groups using the cutoff point as either those who have psychological distress or those who do not [17, 18].

### Statistical analysis

Statistical analyses were performed with SPSS 21.0 for Windows [9]. Data were presented as mean ± standard deviation (SD), as median (minimum-maximum), and as frequency (%). The Shapiro–Wilk test was used to analyze the normality distribution assumption of the quantitative outcomes. Data were analyzed by Student *t*-test and Mann–Whitney U test for normally distributed and non-normally distributed data, respectively. The results were evaluated using the nonparametric Kruskal Wallis test for comparison between groups. The frequencies were compared by using the Pearson Chi-square and Continuity Correction Chi-square. The relation between variables was assessed by Spearman rank correlation for non-normal data. Multivariate logistic regression analyses were performed to identify independent predictors of PTSD status and earthquake injuries. Odds ratios (OR) and 95% confidence intervals (CI) were used to evaluate the risk of variables. A *p*-value less than 0.05 was considered statistically significant.

## Results

In this study, the distribution of earthquake survivors according to earthquake knowledge, attitude, behavior, and experiences was provided in Table 2. Of the survivors, 331 (14.3%) were trapped under debris and 330 (14.2%) were injured, and injuries often occurred during the first earthquake.

**Table 2:** Distribution of the study group according to the questions about the earthquake (*n* = 2317)

		n	%
**First reaction during the earthquake**			
	Stayed in the same place	342	14.8
	Sat down	259	11.2
	Stood up	66	2.8
	Hid under the desk or furniture	112	4.8
	Ran out but trapped	438	18.9
	Ran out of the building	1096	47.3
	Others	4	0.2
**The state of being trapped after the earthquake**			
	No	1986	85.7
	Yes	331	14.3
**Time spent under the debris**			
	0–2 h	151	45.6
	3–8 h	130	39.3
	9–24 h	42	12.7
	2 or 3 days	5	1.5
	4–7 days	2	0.6
	Unknown	1	0.3
**The state of getting injured during the earthquake**			
	No	1987	85.8
	Yes	330	14.2
**When the injury took place**			
	At the time of the first earthquake	311	94.2
	At the 2nd earthquake	16	4.8
	After the earthquake	3	1.0
**Main cause of injury**			
	Falling from height	11	3.3
	Coming in contact with a falling object	182	55.2
	Being trapped under the debris	86	26.1
	Sliding/tripping	49	14.8
	Other	2	0.6
**Injured area of ​​the body**			
	Head	34	10.3
	Neck and trunk	89	27.0
	Hand-arm	61	18.5
	Foot-leg	146	44.2
**Loss of relatives**			
	First degree (Mother. father. spouse. child)	397	17.1
	Second degree (Brother. uncle. cousin. etc.)	1182	51.0
	No loss	738	31.9
**Severity of house damage**			
	None	36	1.6
	Slightly to moderately damaged	434	18.8
	Severely damaged to be demolished	1249	53.9
	Completely destroyed	598	25.8

The relationship between earthquake survivors’ injury status and some demographic characteristics and earthquake-related factors has been shown in Table 3. In the study, the injured and uninjured subgroups were compared in terms of age, marital status and the status of being trapped under the debris, and the *p* values are respectively *p* = 0.016, *p* = 0.001 and *p* < 0.001.

**Table 3:** The distribution of the injury status of the study group according to demographic and earthquake-related factors

			Not injured (*n* = 1987)		Injured (*n* = 330)		
			n	%	n	%	*p*
**Age Groups**							0.016
		≤ 20	91	4.6	8	2.4	
		21–30	310	15.6	60	18.2	
		31–40	425	21.4	50	15.2	
		41–50	422	21.2	70	21.2	
		> 50	739	37.2	142	43.0	
**Gender**							0.793
		Male	1009	50.8	165	50.0	
		Female	978	49.2	165	50.0	
**Marital status**							0.001
		Single	194	9.8	34	10.3	
		Married	1651	83.1	252	76.4	
		Divorced/widowed	142	7.1	44	13.3	
**Educational level**							0.052
		No degree	456	22.9	70	21.2	
		Below high school	1133	57.0	175	53.0	
		High school	282	14.2	54	16.4	
		Above high school	116	5.8	31	9.4	
**Employment status**							0.304
	Unemployed		96	4.8	20	6.1	
	Employed		758	38.1	113	34.2	
	Student		41	2.1	6	1.8	
	Retired/ill/old		254	12.8	54	16.4	
	Housewife		838	42.2	137	41.5	
**The status of having received earthquake evacuation training**							0.487
	No		1932	97.2	318	96.4	
	Yes		55	2.8	12	3.6	
**The status of having been trapped after the earthquake**							<0.001
	No		1896	95.4	90	27.3	
	Yes		91	4.6	240	72.7	

Important factors for the injury status have been evaluated. When compared in terms of participants aged < 20 years of age, the risk of injury was found as OR = 2.74, 95% CI 1.00-7.52, *p* = 0.050 for participants between the ages of 21 and 30; as OR = 1.89, 95% CI 0.66–5.47, *p* = 0.238 for participants between the ages of 31 and 40; as OR = 3.28, 95% CI 1.14–9.48, *p* = 0.028 for participants between the ages of 41 and 50 and as OR = 3.07, 95% CI 1.09–8.61, *p* = 0.033 for participants aged > 50 years of age. When compared in terms of single participants, risk factor was found as OR = 0.70, 95% CI 0.41–1.19, *p* = 0.188 for married participants and as OR = 1.54, 95% CI 0.70–3.37, *p* = 0.284 for participants who were divorced/widowed and when compared with participants who were not trapped under the debris, as OR = 57.00, 95% CI 41.05–79.14, *p* < 0.001 for being trapped under the debris.

While the average GHQ score of survivors was 3.81 ± 2.81, it was found that 51.9% of them were experiencing psychological distress. GHQ-12 scores and statistical evaluations corresponding to these subgroups for the evaluation of the psychological condition of subgroups of participants are shown in Table 4. Being a woman, being a housewife, having been injured, having a loss of first-degree relative, having been trapped under the debris and owning a completely destroyed house were associated with higher level of psychological distress (*p* < 0.001).

**Table 4:** Distribution of GHQ-12 score by demographic and earthquake-related subgroups (*n* = 2317)

		Mean ± SD	Median (Min-Max)	*p*
**Gender**				<0.001
	Male	3.49 ± 2.79	3.00 (0.00–12.00)	
	Female	4.13 ± 2.78	4.00(0.00–12.00)	
**Age groups**				0.797
	≤ 20	3.91 ± 2.70	4.00(0.00–12.00)	
	21–30	3.79 ± 2.67	4.00(0.00–12.00)	
	31–40	3.65 ± 2.79	3.00(0.00–12.00)	
	41–50	3.86 ± 2.80	4.00(0.00–12.00)	
	> 50	3.85 ± 2.89	4.00(0.00–12.00)	
**Marital status**				0.481
	Single	3.83 ± 2.67	4.00(0.00–12.00)	
	Married	3.78 ± 2.82	4.00(0.00–12.00)	
	Divorced/Widowed	3.99 ± 2.77	4.00(0.00–12.00)	
**Educational level**				0.357
	No degree	3.72 ± 2.81	3.00(0.00–12.00)	
	Below high school	3.89 ± 2.84	4.00(0.00–12.00)	
	High school	3.62 ± 2.70	4.00(0.00–12.00)	
	Above high school	3.72 ± 2.69	4.00(0.00–10.00)	
**Employment status**				<0.001
	Unemployed	3.28 ± 2.83	3.00(0.00–12.00)	
	Employed	3.59 ± 2.77	3.00(0.00–12.00)	
	Student	3.45 ± 2.51	4.00(0.00–7.00)	
	Retired/ill/old	3.61 ± 2.84	3.00(0.00–12.00)	
	Housewife	4.13 ± 2.81	4.00(0.00–12.00)	
**The state of getting injured during the earthquake**				<0.001
	No	3.66 ± 2.82	3.00(0.00–12.00)	
	Yes	4.65 ± 2.59	5.50(0.00–12.00)	
**Loss of relatives**				<0.001
	No loss	3.21 ± 2.75	3.00(0.00–12.00)	
	Second degree (Brother, uncle, cousin, etc.)	3.91 ± 2.78	4.00(0.00–12.00)	
	First degree (Mother, father, spouse, child)	4.60 ± 2.73	5.00(0.00–12.00)	
**The status of having been trapped after the earthquake**				<0.001
	No	3.69 ± 2.82	3.00(0.00–12.00)	
	Yes	4.51 ± 2.63	5.00(0.00–12.00)	
**Severity of house damage**				<0.001
	None	2.42 ± 2.64	2.00(0.00–9.00)	
	Slightly to moderately	3.08 ± 2.74	2.50(0.00–12.00)	
	Severely damaged to be demolished	3.84 ± 2.78	4.00(0.00–12.00)	
	Completely destroyed	4.35 ± 2.79	5.00(0.00–12.00)	

To examine factors associated with severe psychological distress, all the significant findings from the univariate analysis were exposed to logistic regression analysis. According to the analysis, losing a second-degree relative (OR = 1.47, 95%CI 1.22–1.78, *p* < 0.001), and losing a first-degree relative (OR = 2.02, 95% 1.55–2.63, *p* < 0.001) correlated with higher psychological distress. All results regarding logistic regression are given in Table 5.

**Table 5:** The results of logistic regression analysis

		OR (95% CI)	*p*
**Gender**			
	Male	1.0 (ref.)	
	Female	1.46 (1.08–1.96)	0.014
**Employment status**			
	Unemployed	1.0 (ref.)	
	Employed	1.08 (0.73–1.62)	0.695
	Student	1.19 (0.59–2.40)	0.634
	Retired/ill/old	0.93 (0.60–1.44)	0.739
	Housewife	1.02 (0.64–1.61)	0.940
**The state of getting injured during the earthquake**			
	No	1.0 (ref.)	
	Yes	1.45 (1.04–2.02)	0.031
**Loss of relatives**			
	No loss	1.0 (ref.)	
	Second degree (Brother, uncle, cousin, etc.)	1.47 (1.22–1.78)	<0.001
	First degree (Mother, father, spouse, child)	2.02 (1.55–2.63)	<0.001
**The status of having been trapped after the earthquake**			
	No	1.0 (ref.)	
	Yes	1.05 (0.74–1.49)	0.795
**Severity of house damage**			
	None	1.0 (ref.)	
	Slightly to moderately	1.67 (0.79–3.63)	0.177
	Severely damaged to be demolished	2.71 (1.28–5.72)	0.009
	Completely destroyed	3.02 (1.40–6.50)	0.005

## Discussion

In this study, the earthquake-related knowledge, attitudes, behaviours, general health and psychological status of the earthquake survivors after the earthquake that took place in Nurdağı District of Gaziantep were determined and the factors affecting these were examined.

More than 90% of the survivors stated that they had not experienced an earthquake before and approximately 3% stated that they had received training on evacuation. It was found that injuries occured at a rate of 14.2%, 94.2% of which took place at the time of the first earthquake and in 55% of these, the reason for injury was coming in contact with a falling object. Injuries of the leg and foot were the most frequent type of injury with 44.2%.

In a large number of studies conducted due to lack of post-earthquake trauma assessments, injury models, the characteristics of referrals for earthquake-related injuries and related health incomes have been evaluated [3]. Humans tend to be worried, scared and overwhelmed especially after an earthquake [10]. Such behavioral reactions can make them more vulnerable to injury [20].

In an earthquake-related study conducted in Italy, multiple injuries were found in 52% of the survivors and the injuries found were mostly in the lower extremity [3]. In another study, 89% of referrals to hospital were due to injuries and 66% of these referrals had fractures. Lower extremity and trunk injuries were found to have a more severe course than head and neck injuries [1]. The rate of injury was 5.6% in the survivors of the earthquake that took place in Lushan [21].

After the Wenchuan 2008 earthquake, it was found that 43.3% of the survivors had one injury, while the rest had two or more injuries and most of the injuries were in the lower extremity (22.9%) and the head (30.8%) [22]. After the earthquake that took place in China, injuries were found most frequently in the head and face (28.5%), followed by the head and the lower extremities, while the fractures were mostly in the lower extremity (35.66%), upper extremity (23.37%) and thorax (17.11%) [23]. In a similar study, post-earthquake injury locations in front-line hospital and second-line hospital were found as lower extremities 23.9–29.2%; the head 26.3–11.1% and trunk 32.5–28.6%, respectively [24]. In an earthquake-related study conducted in Turkey, the first three most frequent injuries related with musculoskeletal trauma were found as soft tissue injuries, followed by fractures and contusion injuries [25]. After the Haiti earthquake, rate of injury determined was 66% and the rate of fracture was 47% [26]. In a study, it was shown that stress affected bone metabolism and increased the risk for fractures [27].

While the injury rates in the present study were found to be lower than most studies, injuries were mostly in lower extremities, the trunk and neck region, similar to other studies conducted. While gender, educational level, occupation and the state of having received training on evacuation did not have any effects on injury; age, marital status and being trapped under the debris were found to have effects on injury. Age groups of 21–30, 41–50 and > 50 and being trapped under the debris were found to be important risks for injury.

In a study evaluating earthquake evacuation, it was stated that 52.16% of the survivors left the building running, while 20.29% stayed in the same place. Injury rates were found to be higher in survivors who were trapped under the debris and those who were older than 65 years of age [21]. While the rate of leaving the building was lower in this study, injury rate was found to increase with the increase in age and being trapped under the debris. In literature, the first reactions after the earthquake and fears during the earthquake were found to be correlated with injury. In other words, correlation was shown between individual behavioral characteristics and earthquake-related injury [21].

It was found that 61.05% of the survivors in Lushan had received training on earthquake evacuation and the rate of having received evacuation training was 47.59% in those who were injured and 61.85% in those who were not [21]. The rate of having received earthquake evacuation training was very low in our study, while there was no statistical difference between survivors who had received training and those who had not.

In terms of having losses in the family, it was found that 17.1% of the survivors had lost first degree relatives, while 51.0% had lost their second degree relatives. The rate of survivors whose houses were destroyed or were about to be destroyed was in the majority (53.9%). Mean GHQ-12 score of the survivors was 3.81 ± 2.81 and it was found with GHQ-12 that 51.9% experienced psychological distress. It was found that women, housewives, those who were injured, those who lost their first degree relatives, those who were trapped under the debris and those whose houses were destroyed experienced more psychological distress. At the same time, while mean GHQ-12 scores of the survivors who were younger than 20 years of age, those who were widowed and those who had received education below high school were higher than the mean scores of the survivors in their categories, no statistically significant difference was found.

In the evaluation with logistic regression in terms of psychological stress, being female, having been injured in the earthquake, loss of first-degree relatives, loss of second-degree relatives, having a severely damaged-to be demolished house and having a completely destroyed house were found to be associated with higher psychological stress.

In a study conducted on earthquake survivors, it was found that 40.1% of the survivors had post traumatic stress disorder 1 year after the earthquake, while a meta-analysis report found post-traumatic stress disorder with a rate of 29% in the 9-month period following the earthquake among 76,101 individuals [7]. In Bam province of Iran, psychological distress was measured with GHQ-12 and mental problems were found with a rate of 58%. More severe psychological distress was reported in individuals who were older, females, individuals with lower educational level, divorced or widowed and unemployed participants and participants who lost more family members in the earthquake [6]. In adolescent survivors who were affected by the Wenchuan earthquake, PTSD prevalence was found as 5.7% and injury prevalence was found as 8.8%, as measured by various scales. Positive risk factors of PTSD include losing house and property, being injured, death of family members and witnessing death, while negative risk factors are social support and physical exercise [28]. In another study, delayed-onset PTSD symptoms prevalance was found as 9.7% as a result of 7-year long follow up in 340 survivors. While being injured during the earthquake, being unemployed and social support were found to be significant for PTSD symptoms in univariate analysis; unemployment was found to be a risk factor and social support was found to be a protective factor for PTSD symptoms in multivariate analysis [7]. In the literature, psychological stress was found to be 15.4% with the Professional quality of life (ProQOL) scale as a result of seven years of follow-up after earthquake [29]. The relationship between age and posttraumatic stress was evaluated in survivors of Sichuan earthquake and PTSD and general psychiatric morbidity were found to be more frequent in elders when compared with young individuals. Significant correlations were found between PTSD development and risk factors of being old, having been in serious danger, loss of family members and guilt related with one’s death or injury [30]. In a study, GHQ-12 scores were found to be significantly higher at most stressful times and even five months after the earthquake. It was shown that increase in age had an effect on GHQ-12 scores of the subjects [16].

It was also found that in addition to total GHQ score, advanced age also had an effect on the impaired recovery of factor ‘social dysfunction’ score [17]. In the assessment of the psychologies of survivors with different scales, the prevalence of PTSD was found as 40.1%. Being a woman, having a low level of income and a low level of perceived social support were found to be risk factors associated with anxiety, depression and PTSD [31].

In the first month following the L’Aquila earthquake, risk factors of patients diagnosed with PTSD were examined. The fear of having aftershocks all the time and impairment of working memory backward were found to be risk factors [32]. After the earthquake that occurred in Italy, PTSD prevalence was found as 14.5% and male gender, age under 55 years, and better school education were found to be protective factors for PTSD [33]. In another study, PTSD prevalence was found as 9.2% with PTSD checklist-civilian version questionnaire. Multiple logistic regression analysis showed females, elders, farmers and those who lost a family member were riskier for PTSD development [34].

When the effect of earthquake on adolescents was evaluated, while economic loss, separation from parents, and worrying about future aftershocks were risk factors for PTSD, good social relationships and positive news were protective factors [35, 36].

The present study and similar studies evaluated PTSD prevalence and the affecting factors. PTSD prevalence has often been found with GHQ-12 and sometimes by using different scales. Similar to the results of our study, it has been found that female gender, being a housewife, being trapped under the debris, being injured in the earthquake and loss of relatives have significant effects on psychological distress, while unlike our study it was found that having low income, being widowed, unemployed and older increased stress. Also better education, doing exercise after the earthquake and receiving social health were protective factors for PTSD.

As a conclusion, injuries were mostly in the lower extremity, trunk and neck area in the present study and increased age and being trapped under the debris were important risks for injury. It was found that more than half of the survivors had psychological distress, while female gender, being injured in the earthquake, and losing a first degree relative had higher psychological distress, while loss of first and second degree, having a severely damaged house and having a completely destroyed house were associated with higher levels of psychological distress.

Some limitations are present in this study on earthquake survivors. The first of these was that while the earthquake was seen in a much larger region, the study was conducted only in Nurdağı and the participants were earthquake victims residing in Nurdağı. However, it must be noted that the Nurdağı District was one of the residential places where the earthquake was experienced most severely. The study was obtained from the results of a survey conducted 2 months after the earthquake, and the psychological states of the survivors were evaluated with the GHQ-12 scale. This scale is a method used to measure mental health in general, and no different method has been used to evaluate this situation. In this study, the mental health of the survivors was evaluated shortly after the earthquake, and the long-term effect of the earthquake on mental health was not examined. Furthermore, the study group only consisted of earthquake survivors and a control group to compare the effects of the earthquake was not included in the study.

In this study, the general and mental health of earthquake victims was examined and its interaction with different factors was shown, and in this respect it will make a significant contribution to the literature. Since the first-reaction after an earthquake carries vital importance in the prevention of injuries, sharing experiences and educational information related to earthquakes may prevent injuries; keeping in mind that psychological counseling after an earthquake can reduce the negative effects on mental health, it is necessary and essential that such forms of support be provided.

## Conclusions

Medical data for finding out the health status of earthquake survivors are important sources of information. However, there are problems in obtaining data due to errors and deficiencies as a result of the chaotic situation in earthquake regions. The effects of trauma, interacting factors and different individual behaviors of earthquake survivors are ignored in studies. The present study is a large-scale study conducted on survivors of the earthquake in Nurdağı District and has shown demographic features, earthquake-related issues and health status. Specific follow-up studies such as longitudinal evaluations, studies on earthquake survivors in other locations, and behavioral interventions that can set forth the general and mental health status of earthquake survivors can be made to alleviate the destructive impact of earthquakes on humans.

## Data Availability

The datasets used and/or analysed during the current study available from the corresponding author on reasonable request.

## Abbreviations

GHQ-12: The 12-item General Health Questionnaire
PTSD: Post-traumatic stress disorder
ProQOL: Professional quality of life
SD: Standard deviation
OR: Odds ratio
CI: Confidence interval
